# Functional MRI Evaluation of Multiple Neural Networks Underlying Auditory Verbal Hallucinations in Schizophrenia Spectrum Disorders

**DOI:** 10.3389/fpsyt.2016.00039

**Published:** 2016-03-29

**Authors:** Robert. J. Thoma, Charlotte Chaze, Jeffrey David Lewine, Vince D. Calhoun, Vincent P. Clark, Juan Bustillo, Jon Houck, Judith Ford, Rose Bigelow, Corbin Wilhelmi, Julia M. Stephen, Jessica A. Turner

**Affiliations:** ^1^Department of Psychiatry, University of New Mexico, Albuquerque, NM, USA; ^2^The Mind Research Network and the Lovelace Family of Companies, Albuquerque, NM, USA; ^3^Department of Psychology, University of New Mexico, Albuquerque, NM, USA; ^4^Department of Electrical and Computer Engineering, University of New Mexico, Albuquerque, NM, USA; ^5^Department of Psychiatry, University of California San Francisco School of Medicine, San Francisco, CA, USA; ^6^Department of Psychology, Georgia State University, Atlanta, GA, USA

**Keywords:** auditory verbal hallucinations, schizophrenia, functional magnetic resonance imaging, independent component analysis, general linear model

## Abstract

Functional MRI studies have identified a distributed set of brain activations to be associated with auditory verbal hallucinations (AVH). However, very little is known about how activated brain regions may be linked together into AVH-generating networks. Fifteen volunteers with schizophrenia or schizoaffective disorder pressed buttons to indicate onset and offset of AVH during fMRI scanning. When a general linear model was used to compare blood oxygenation level dependence signals during periods in which subjects indicated that they were versus were not experiencing AVH (“AVH-on” versus “AVH-off”), it revealed AVH-related activity in bilateral inferior frontal and superior temporal regions; the right middle temporal gyrus; and the left insula, supramarginal gyrus, inferior parietal lobule, and extranuclear white matter. In an effort to identify AVH-related networks, the raw data were also processed using independent component analyses (ICAs). Four ICA components were spatially consistent with an *a priori* network framework based upon published meta-analyses of imaging correlates of AVH. Of these four components, only a network involving bilateral auditory cortices and posterior receptive language areas was significantly and positively correlated to the pattern of AVH-on versus AVH-off. The ICA also identified two additional networks (occipital–temporal and medial prefrontal), not fully matching the meta-analysis framework, but nevertheless containing nodes reported as active in some studies of AVH. Both networks showed significant AVH-related profiles, but both were most active during AVH-off periods. Overall, the data suggest that AVH generation requires specific and selective activation of auditory cortical and posterior language regions, perhaps coupled to a release of indirect influence by occipital and medial frontal structures.

## Introduction

Auditory verbal hallucinations (AVH) are a common positive symptom in schizophrenia spectrum disorders (SSD), and they can be functionally disruptive for individuals who experience them. Available data indicate that between two thirds and three quarters of individuals who hear voices become depressed and disturbed by the experience (1–5). Individuals with AVH are more likely to develop post-psychotic depression, feelings of loss, humiliation, and entrapment. The presence of AVH has also been associated with a higher risk for suicide (6). Treatment with antipsychotic medication can usually reduce the severity and frequency of AVH (7), but the high prevalence of relapse and recurrence of psychosis (estimated at 81.9% within 5 years) (8, 9) and the ubiquity of medication side effects necessitate the development of new approaches for treatment of AVH. Novel, directed, and neuromodulatory treatments for AVH, such as transcranial magnetic stimulation (TMS) and transcranial direct current stimulation (tDCS) (10), are presently under development and would benefit from a more detailed understanding of the neural circuitry underlying AVH (10, 11).

Across structural (12–14) and functional MRI (15–17) studies, auditory cortex and speech processing regions of the posterior superior temporal cortex and inferior prefrontal cortex are frequently implicated as critical substrates underlying AVH generation. Beyond these core auditory and language regions, activations in other areas have been reported only inconsistently. To evaluate the problem of variability across functional imaging studies of AVH, Jardri and colleagues performed a coordinate-based meta-analysis of fMRI and PET studies of patients with AVH (15). The authors identified reliable AVH-related clusters within a bihemispheric neural network that included the anterior insula and frontal operculum in the right hemisphere and Broca’s area, anterior insula, precentral gyrus, middle and superior temporal gyri, inferior parietal lobe, and hippocampus and parahippocampal regions in the left hemisphere (15). Table 1 presents a summary of recent meta-analyses addressing regional activations associated with AVH.

**Table 1:** **Review of the findings of three quantitative meta-analyses of AVH-related activations**.

Jardri et al. (20)	Brodmann
Cluster A: Broca’s convolution, left anterior insula, and left precentral gyrus	44, 13, 6
Cluster B: left hippocampus/parahippocampal gyrus	27
Cluster C: right anterior insula and right frontal operculum	13, 47
Cluster D: superior and middle temporal gyri	21, 22
Cluster E: supramarginal gyrus	40

**Kuhn and Gallinat (22)**	**Brodmann**

Left parietal operculum	43/40
Left postcentral gyrus	3
Right postcentral gyrus	3
Left inferior frontal gyrus	44

**Lutterveld et al. (21)**	**Peak coordinates**

Putamen extending into insula and precentral gyrus (left)	−44, 0, 6
Insula (right)	53, 11, −4
Postcentral gyrus (left)	−47, −17, 46
Pulvinar (thalamus) extending into the claustrum (left)	−30, −29, 6
Medial frontal gyrus (right)	6, 6, 61
Culmen (right)	20, −54, −21
Inferior frontal gyrus (pars opercularis; right)	60, 8, 12
Inferior frontal gyrus (pars orbitalis; right)	48, 24, 0
Medial geniculum body (left)	−16, −24, −4
Insula (left)	−55, −19, 16
Postcentral gyrus (right)	56, −16, 20
Insula (left)	−48, −40, 24
Postcentral gyrus (right)	64, −16, 36
Postcentral gyrus (right)	60, −24, 44
Claustrum (right)	40, −4, 4
Insula (right)	44, 16, 10
Superior temporal gyrus (left)	−60, −56, 20
Postcentral gyrus (left)	−60, −20, 40
Precentral gyrus (left)	−50, 6, 33
Parahippocampal gyrus (left)	−24, −33, −6
Claustrum (right)	28, 27, −5
Pyramis (right)	20, −64, −30

The present study used a within-subject approach in which subject’s self-defined periods with active AVH (that is, “AVH-on” periods) and periods without AVH (that is, “AVH-off” periods), as delineated by button presses signifying the onset versus offset of AVH. A general linear model (GLM) comparing AVH-on versus AVH-off periods was performed, in part, so that the current data could be assessed within the context of the majority of prior fMRI studies of AVH. GLM analysis identifies brain regions that show activity profiles correlated to the AVH-on versus AVH-off time course, and it was anticipated that findings in the current study would be consistent with prior studies.

Information on which brain nodes are active during AVH is of great interest, but a detailed understanding of how these nodes interact to form AVH-related networks is needed to better understand the neurobiology of AVH generation. That is, to understand how *integrated neural network* activity underlies AVH, there is a need to analyze functional coherence between brain regions. Toward this end, the present study used the well-established method of independent components analysis (ICA) to identify AVH-related neural networks (18). In brief, based upon findings that distinct brain regions exhibit synchronous fluctuations in intrinsic activity, ICA has become a common technique for measurement of functional connectivity between brain regions (18). ICA was developed to solve problems similar to the “cocktail party” scenario, in which spatially independent voices must be resolved from microphone recordings of many people speaking at once (19). When applied to fMRI, ICA assumes the presence of spatially independent brain networks, each with associated time courses. These spatial components identify temporally coherent networks [TCN (18)]. The empirical derivation of ICA components is less prone to bias than other analytic approaches since it requires few constraint conditions and little investigator input during analytic processing (18). As the present study used a within-subjects approach in which AVH-on periods were analyzed relative to periods of AVH-off, a data-driven approach, such as ICA, is expected to be maximally sensitive to subtle differences between AVH-on and AVH-off periods.

Using an ICA approach, it was anticipated that AVH-related linking profiles between nodes (that is integrated networks) could be extracted from the data, with an expectation that such network analyses would lead to a more restricted picture of the brain activity profile responsible for generation of AVH. In particular, it was hypothesized that coordinated activity involving auditory cortex and language areas would be a key substrate of AVH.

## Materials and Methods

### Participants

The study was approved by the University of New Mexico Health Sciences Center, Human Research Protections Office, and all participants provided written informed consent. Participants for this study were recruited through flyers posted around the Albuquerque metropolitan area, physician referrals, and direct recruitment of participants from previous studies. Each participant was assessed with an initial brief screening to determine their suitability for the study. After screening, DSM-IV diagnoses of schizophrenia or schizoaffective disorder were determined using the SCID-I/P for DSM-IV-TR, Patient Edition (20). Taking a dimensional approach to SSD (21), patients meeting criteria for both diagnostic categories were included in the study as there is no reason to predict differences in rates or types of hallucination between diagnoses (22). Exclusionary criteria included history of head injury with loss of consciousness or other neurological disorder, diagnosis of mental retardation, and current substance abuse or dependence (except for nicotine). Head injury, neurological disorder, and mental retardation were determined using a questionnaire developed for this study and substance dependence with the SCID-I/P (20). Additionally, a urine drug screen and breathalyzer were administered to all participants on the morning of the examination to assure that all were substance free at the time of scanning and other data collection. If participants had positive results for any drugs of abuse or alcohol, they were excluded from study participation. All female participants were additionally screened for pregnancy immediately prior to MRI scanning. A total of nineteen volunteers (ages 20–60 years; 11 males/8 females) met all study criteria and participate in all study procedures.

### Behavioral Assessment Procedures

Beginning the 2 weeks prior to neuroimaging, each participant completed an “AVH diary,” recording the frequency, intensity, and content of their AVH. This information was used to determine the likelihood that they would experience AVH during scanning and to find the best time of day for individual scanning. On the day of neuroimaging, participants were administered the Positive and Negative Symptoms Scales [PANSS (23)] and the Psychotic Symptom Rating Scales [PSYRATS (24)] as part of a larger diagnostic battery of measures. Brief pre- and post-scan semistructured interviews were also administered to record their levels of comfort, anxiety, and stress, and to gather qualitative data regarding phenomenology of their AVH experience during neuroimaging data collection. The post-scan interview was also designed to assess and record any difficulties participants had with the button-press task or other procedures.

### Button-Press Procedures

Pre- and post-study procedures were undertaken to assure the validity of button presses as true indicators of AVH-on and AVH-off. First, during the week prior to scanning, each subject participated in one or more “button press” training sessions. During these sessions, the investigative team carefully discussed with each subject what to consider as an AVH and how and when to push buttons indicating AVH onset versus offset. Subjects were trained to push (and immediately release) a button with their right hand to indicate AVH onset and a button with their left hand to indicate AVH offset. Practice on button-press procedures was also administered immediately prior to scanning and immediately following the pre-scan interview.

During the imaging component of the study, participants were continuously monitored by research staff. Voice communication was possible at all times, so if the staff suspected that the subject was not accurately reporting AVH (e.g., as indexed by multiple AVH-on button presses in a row, in the absence of any AVH-off button presses), this could be documented and the subject could be redirected. Appropriate sequencing of AVH-on and AVH-off button presses was rechecked prior to additional data processing, with rejection of periods with improper sequencing (e.g., three on presses followed by an off press). Sequencing errors were rare, which is an indication that subjects mostly understood and properly performed the task.

### MRI Procedures

Participants were scanned using a 3-T Siemens TIM Trio MRI at the Mind Research Network (MRN), Albuquerque, NM, USA. The scanning protocol included a localizer, a T1-weighted anatomical scan, and multiple resting-state functional scans. T2-weighted EPI images were acquired with an interleaved cycle of 33 slices (3.5 mm thickness, 1.05 mm gap) of 3.8 mm × 3.8 mm × 3.5 mm voxels, leaving a total field of view of 240 mm. TE and TR were set at 29 ms and 2 s, respectively, with a flip angle of 75° and a 64 × 64 matrix, with a total of 150 TRs in each resting-state run. Subjects were scanned with their eyes open while looking at a fixation cross with a button box in each hand. Eye movements were tracked to assure that participants were alert and attending to task demands during scanning. Button-press information was recorded using Presentation.^1^ A minimum of three 5-min scan runs were collected per subject. If a given subject did not have any hallucinations during that 15-min period, additional 5-min scan runs were collected, as needed.

### MRI Preprocessing

Data preprocessing was performed using SPM5^2^ in an automated analysis pipeline developed at MRN (25). This processing pipeline included removal of the first three images for T1 saturation effects, slice timing correction, and motion realignment to the middle frame of each 5-min scan using INRIAlign (26), normalization to MNI space (27), reslicing to 3 mm × 3 mm × 3 mm voxels, and smoothing using a 10-mm full-width at half maximum kernel (FWHM). Subsequent to automated preprocessing, the data were intensity normalized to improve the accuracy and test–retest reliability of independent components analysis (ICA) output (28). Intensity normalization divides the time series of each voxel by its average intensity, converting data to percent signal change units. All data were additionally subject to analysis-specific outlier detection procedures.

### General Linear Model

Two subjects were identified by GLM outlier detection procedures as having beta values more than 3 SDs from the group mean, so they were removed from further GLM analyses (outliers are identified in Table 2). For the univariate voxel-wise analysis, each subject’s images, reconstructed without artifacts, were entered into the first level GLM in SPM8, using the AVH onsets and durations in a block design. This first-level analysis provided contrast maps for increased blood oxygenation level dependence (BOLD) signal during the reported AVH, across all scans for each subject. The second-level GLM analysis was done in the SPM toolbox SnPM^3^ for statistical non-parametric mapping of the data. A non-parametric approach was used for the group analysis to best account for the variability in this small sample (29). Correct for multiple comparisons was made in the SPM analysis using an FDR cluster level correction approach.

**Table 2:** **Participant demographic and diagnostic information**.

Subject	Primary diagnosis	Age	Gender	Handedness	Duration of illness (years)	Medication^a^ (dose^b^)
1	Schizophrenia	28	M	R	11	Zyprexa (20.0)
2	Schizoaffective	33	F	R	17	Abilify/zyprexa (2.5)
3	Schizophrenia	39	M	R	12	Clozapine/abilify (30)
4	Schizoaffective	53	F	R	30	Clozapine (6.3)
5	Schizophrenia	55	M	R	32	Abilify/geodon (16.0)
6	Schizophrenia	34	F	R	17	Haloperidol (6.67)
7	Schizophrenia	55	F	R	18	Perphenazine^c^
8	Schizophrenia	39	M	R	5	Clozapine (6.7)
9	Schizophrenia	46	M	R	26	Clozapine (6.7)
10	Schizophrenia	60	M	L	41	Olanzapine (10.72)
11	Schizophrenia	24	M	R	7	Haloperidol/risperidone (10.72)
12	Schizophrenia	37	F	Ambi	13	Abilify (6.7)
13	Schizoaffective	53	F	R	34	Abilify (20.1)
14	Schizophrenia	33	M	L	0.58	Risperidone/abilify (23.39)
15	Schizoaffective	51	M	R	28	Risperidone (9.36)

*^a^Primary and secondary antipsychotic medication*.

*^b^Dosages reported in total olanzapine milligram per day equivalent*.

*^c^Dosage information was not available for this participant*.

### Independent Component Analysis

Identification of networks with ICA is a multistep procedure. In general, ICA is applied to fMRI data to identify maximally spatially independent patterns. ICA was performed using the GIFT software^4^ on processed fMRI images (29). A principal component analysis (PCA) was first used for data reduction, and 100 principal components were retained at level one (30). The second group-level PCA then reduced this to 40 components, the number of components estimated using a modified minimum description length tool built into GIFT (31). This was followed by ICA using the Infomax algorithm, which included ICASSO with 20 repetitions, selecting the most representative run to ensure a stable solution. Components containing obvious artifact were identified based upon spatial location and/or spectral composition. Subject time courses and maps were generated using the back reconstruction (30) as part of GICA (19). Of the array of 40 components, 12 were rejected from further analyses because they appeared to be related to head movement or had inappropriate localization (e.g., within the ventricles). Each subject’s time course for each component from each scan was then regressed against their reported time course of AVH onsets and durations convolved with a canonical hemodynamic response function (HRF). As there were multiple scan runs for each subject, the beta values for these runs were averaged to provide one beta value per individual per component.

Prior to the analyses, it was recognized that our sample size was relatively small and that it would be unlikely that component testing would reveal beta weights with sufficiently large scores to survive statistical correction for multiple comparisons. Therefore, we put into place a component selection/reduction strategy, in which we would only evaluate the significance of that subset of components with all nodes implicated in one or more of three published meta-analyses of AVH (see Table 1).

## Results

Of 19 participants enrolled in the study, 4 were excluded from further analysis because they did not experience AVH during scanning or did not clearly indicate AVH with button presses during scanning. Ten males and five females were included in subsequent analyses, with a mean age of 42.13 years (SD = 11.75). Table 2 presents diagnostic and demographic data for each participant. The primary diagnosis for all subjects was either schizophrenia (*N* = 11) or schizoaffective disorder (*N* = 4). The duration of illness ranged from about 6 months to 41 years, with a mean of 20.1 years (SD = 11.6).

The diagnostic groups of schizophrenia and schizoaffective did not differ on age (*p* = 0.71, partial eta-squared = 0.01) or handedness (*p* = 0.56, partial eta-squared = 0.03), but male gender was overrepresented in the schizophrenia group (8/10) relative to the schizoaffective group (1/5; *p* = 0.02, partial eta-squared = 0.33). Item #3 of the PANSS ranged from 3 (mild; “one or two clearly formed but infrequent hallucinations…”) to 6 (severe; “hallucinations are almost continuously present causing major disruption of thinking…”), with a mean value of 4.6 (SD = 0.7).

As presented in Table 3, hallucinatory modalities included auditory (*N* = 15), visual (*N* = 12), tactile (*N* = 8), olfactory (*N* = 17), and gustatory (*N* = 2). The number of voices heard ranged from 1 to “too many to count.” No difference was found between diagnostic groups (schizophrenia/schizoaffective) on clinical variables, including PANSS item 3 score (*p* = 0.57, partial eta-squared = 0.03), PSYRATS total score (*p* = 0.37, ­partial eta-squared = 0.06), occurrence of visual (*p* = 0.74, partial eta-squared = 0.009), or number of modalities (*p* = 0.18, partial eta-squared = 13).

**Table 3:** **AVH characteristics by participant**.

Subject	PANSS Q3 rating	PSYRATS total	During scan?		Ever?	
			AVH	Visual H	Modalities	# of voices
1	4	22	Yes	Yes	A, O	2
2	4	24	Yes	No	A, V, O	4
3	n/a	25	Yes	Yes	A, V	+
4	5	35	Yes	Yes	A, V, T, O	3
5	3	27	Yes	Yes	A, V, T, O	+
6	5	27	Yes	Yes	A, V, T, O, G	1
7	5	36	Yes	No	A, V, T, O	NR
8^a^	4	37	Yes	No	A, V, T, O	3
9	6	24	Yes	Yes	A, V	7
10	4	27	Yes	Yes	A, V	4
11	5	30	Yes	Yes	A, V, T, O	10
12	5	32	Yes	Yes	A	+
13	5	36	Yes	No	A, V, T	+
14^a^	5	29	Yes	No	A, V, T, O, G	3
15	5	29	Yes	No	A	+

*^a^Participant was included in the ICA analysis but removed from the GLM*.

*Modalities: A, auditory; O, olfactory; V, visual; T, tactile; G, gustatory; NR, no response from participant*.

For each subject, information regarding the number of collected 5-min scan runs, and the frequency and duration of AVH are presented in Table 4. The mean number of scan runs was 4.13, with a SD of 1.

**Table 4:** **fMRI behavioral data**.

Subject	# Scanning sessions	Mean onsets	Mean duration (s)	Min/max duration (s)
1	3.00	7.33	5.08	0.30/26.90
2	3.00	8.33	6.31	0.26/38.04
3	5.00	3.00	43.17	4.55/230.92
4	6.00	8.33	1.49	0.00/13.88
5	3.00	6.33	38.44	3.87/230.92
6	4.00	3.50	31.60	2.24/147.39
7	3.00	5.00	10.42	0.66/54.57
8	4.00	6.75	29.15	4.79/76.11
9	4.00	2.67	0.15	0.07/0.29
10	5.00	1.00	227.70	50.91/298.02
11	4.00	4.50	17.30	3.52/65.63
12	5.00	7.75	2.45	0.31/5.89
13	5.00	2.75	69.87	7.08/133.21
14	4.00	5.50	30.14	6.36/102.86
15	4.00	4.75	3.78	1.13/6.36

Total	62.00	308.00	5898.70	
Mean	4.13	4.97	19.15	
SD	1.00	2.31	42.43	

### General Linear Model Results

**Figure 1:**
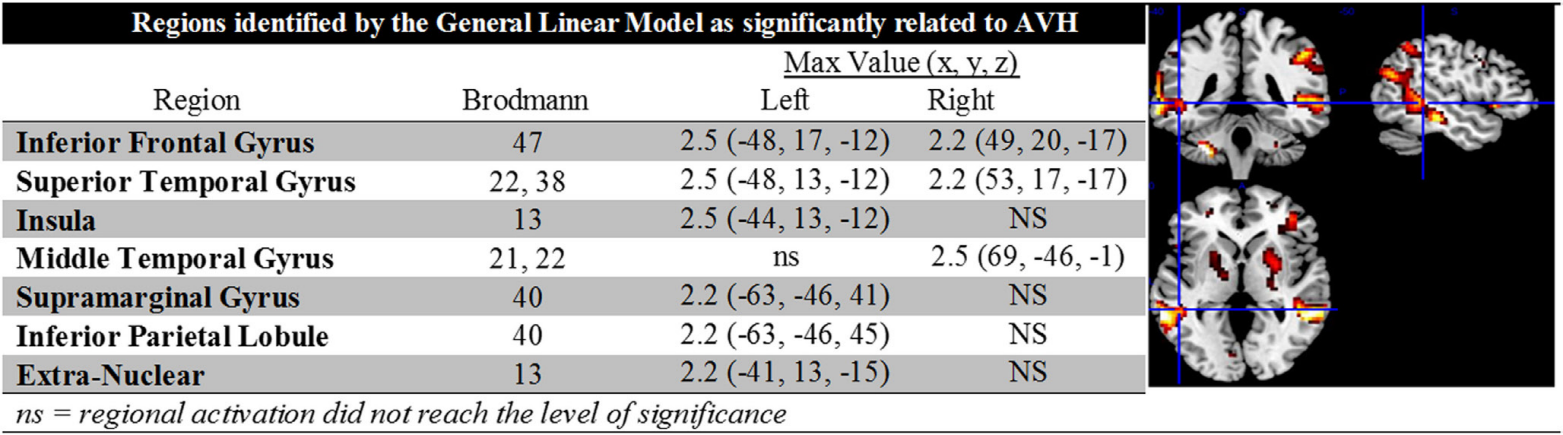
**The figure contains descriptive data and depictions of regional activations identified in the general linear model analysis**. Note the presence of bilateral activations in inferior frontal gyrus and STG, left lateralized activations in the insula, supramarginal gyrus, inferior parietal lobule and extra nuclear cortex, and right lateralized activation in middle temporal gyrus.

The second-level GLM analysis revealed significant increases at the cluster level as shown in Figure 1. The largest regional activations were in inferior frontal gyrus (Brodmann’s area 47), STG (Brodmann’s areas 22 and 38), and supramarginal gyrus (Brodmann’s area 40). Figure 1 contains a complete description of regional activations and a graphic representation projected into MNI space. When entered into the GLM, the effect of diagnosis (schizophrenia/schizoaffective) did not approach a level of significance.

Head motion is a potentially complicating factor in fMRI studies. In the present case, head movement was physically limited by the headphones used for noise control and communication with research staff. Nevertheless, to evaluate whether analyses were impacted by even small task-related head motion, the correlation between motion and the SPM design matrix was computed. Results indicated that there was no significant impact of motion (*p* > 0.8).

### Independent Component Analysis Results

**Figure 2:**
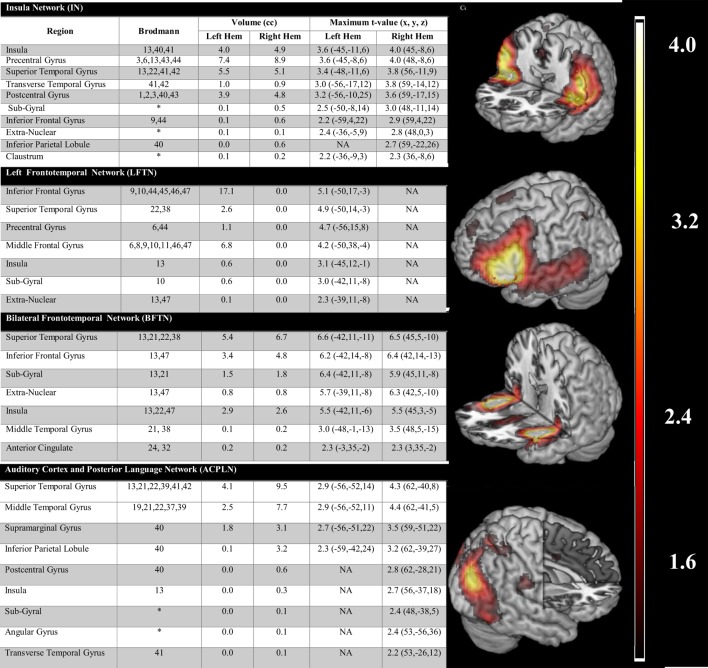
**The figure lists the component activations associated with each network and presents depictions of the regional activations associated with each**.

Only ICA networks that were spatially consistent with the findings of three previously published quantitative meta-analyses (16–18) were kept for further consideration (see Table 1). Only four ICA networks were identified as matching this *a priori* framework. Figure 2 lists the component activations associated with each network and presents depictions of the regional activations associated with each. One sample *t*-tests (corrected for four multiple comparisons) were used to determine the valence and extent of network activation as it related to periods during which participants were experiencing AVH (AVH-on) relative to periods during which they were not experiencing AVH (AVH-off) or the AVH-on/AVH-off temporal time course. Of the four networks, only an auditory cortex-posterior language network (ACPLN) was found to be significantly and positively related to AVH-on and AVH-off [*t*(12) = 3.41, *p* = 0.005]. The insula network (IN; *p* = 0.93), the left frontotemporal network (LFTN; *p* = 0.25), and the bilateral frontotemporal network (BFTN; *p* = 0.88) failed to show activity profiles significantly related to the AVH-on/AVH-off time course.

**Figure 3:**
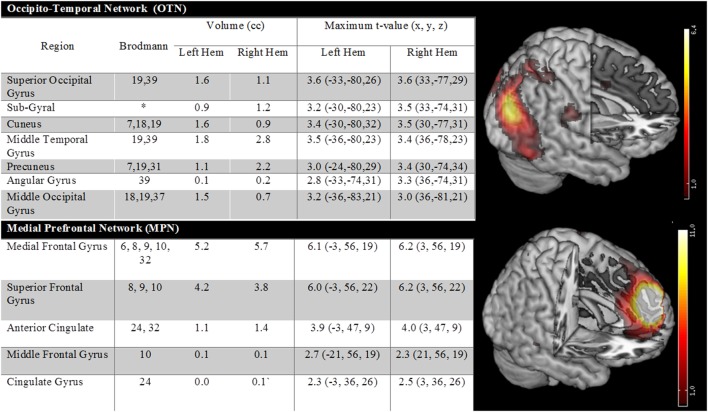
**The figure shows two additional ICA-identified networks which were reported in at least one prior fMRI study**. One of these components involved an occipital temporal network, whereas the other involved medial prefrontal regions.

There were two additional ICA identified networks that had nodes, which had been reported by at least one prior AVH fMRI study (but not showing consistent activation in the meta-analyses). One of these components involved an occipital temporal network, whereas the other involved medial prefrontal regions. Details are provided in Figure 3. When these networks were also considered, they did show significant beta weights [OTN: *t*(12) = −3.01, *p* = 0.01 and MPN: *t*(12) = −3.13, *p* = 0.009]. However, the average beta weights in each case were negative. This suggests that activity profiles in these networks were anticorrelated with the AVH on/off profile, that is, network nodes were more active during AVH-off versus AVH-on periods.

## Discussion

Our approach to understanding AVH networks involved two very different analyses (GLM and ICA) of the same data. Our GLM results generally conformed to the findings of quantitative meta-analyses (16–18), suggesting that our procedures, participants and analyses were consistent with the body of AVH neuroimaging literature. Group-level analyses indicated AVH-related regional activations in the left and right inferior frontal and superior temporal gyri; the right middle temporal gyrus; and the left insula, supramarginal gyrus, inferior parietal lobule, and extranuclear white matter. Unfortunately, this pattern of activity can be construed as consistent with any of the theoretical models of AVH, since it contains activity in at least some of the nodes postulated by each.

The second set of analyses involved independent components analysis (ICA) with a restricted sub-set of components selected for analysis, based upon an *a priori* framework derived from prior meta-analyses (16–18). Given the small sample size, this hypothesis/framework driven approach was needed to restrict the number of multiple comparisons.

In this analysis, only activity in the ACPLN network involving auditory cortex (e.g., Brodmann’s areas 41, 42, 24, 21, and 22) and posterior language regions (Brodmann’s areas 39 and 40) showed a *significant* positive correlation with the on–off profile of AVH. Of the many non-exclusive theoretical models regarding the origins of AVH in SSD that have been proffered in the past, the present results are thought to be most consistent with the set of models, suggesting that AVH arise as a consequence of aberrant activity in auditory cortical networks [i.e., Ref. (32, 33)]. It is also perhaps significant that all four networks, though not all significantly correlated to the AVH-on/AVH-off pattern, all had significant regional activations of superior temporal gyrus auditory cortical regions. It may also be useful to consider this finding in the context of evidence regarding the timing of these activations, derived from magnetoencephalographic (MEG) data collected in our lab (Lewine et al., under review^5^). Analysis of the MEG data showed that transition into the AVH-on state was simultaneous with the onset of transient, abnormal activity high-theta/low alpha band activity generated in auditory cortex and associated receptive language areas of the planum temporal. Our developing theory, based upon all of these data, is that abnormal AVH-related activation in auditory cortex results in erroneous placement of a “stamp,” indicating of external generation on internal speech such that this internal dialog is perceived as having an outside origin. That said the present findings are not inconsistent with the second set of models that suggest that inner speech is erroneously assigned to an external source due to failure frontotemporal circuitry involved in predictive coding (34) or in corollary discharge (35). In these models, abnormal functioning of circuits underlying coordination of sensory motor information during perception results in the inappropriate perception of voices. Indeed, our prior research indicates that persistent, aberrant, inferior frontal theta-band signal seen across patients with schizophrenia (not just those with AVH) may also play a role in AVH generation.

Based on prior research, it was somewhat surprising that networks with primary nodes in inferior frontal regions (LFTN and BFTN) or the insula (IN) did not show activity profiles significantly correlated to the time course of AVH. This does not appear to be a power problem, as correlations were not even close to significant (*p* > 0.5). The apparent discrepancy between GLM and ICA results may reflect the fact that, while activation in inferior frontal and insula regions is increased overall during AVH, such activation is not obligatory for the generation of AVH. It is also notable that ICA also revealed occipital–temporal and medial prefrontal networks that contained brain regions active in some prior imaging reports (but not surviving the meta-analyses). Activity in these networks did show some correlation with the AVH pattern, but beta weights for both were negative. This suggests that these networks switched into an “off” state when AVH emerged. The medial prefrontal cortex is believed to be involved in high level executive functioning and decision making processing, so perhaps hallucinations can only emerge when normal executive functioning is partly “offline.” The second possible conclusion from this finding is that since medial prefrontal cortex is a key node in the default mode network and in ventral attention networks (36), the experience of AVH may represent a transition from a state of resting attentiveness to a network pattern more closely associated with perceptual experience. The potential role of offset of the occipital–temporal network in AVH is less certain, but it might relate to a needed release of visual control of the middle temporal gyrus for generation of AVH. Future, larger studies able to explore cross-coupling between networks may help to clarify this situation.

The present analyses were inconclusive with regard to a third set of theoretical models which regard dysfunction in the circuitry regulating episodic verbal memory as primary in AVH generation. These memory-based models of AVH postulate that hallucinations are a product of misremembered episodic memories of speech (37). The present GLM analysis showed clear activation of right middle temporal gyrus, which might be interpreted as activation of memory-related cortical regions lending support to memory models. Conversely, there was no evidence of medial temporal cortex activation in any of the AVH-related networks. Hence, we conclude that while our fMRI data are generally consistent with current models of AVH generation, for distinction between alternative models, there is a need to have more explicit and fine grain information on the exact temporal sequence in which brain structures are recruited immediately before and after the onset of AVH. Extraction of such information may be possible through on-going studies of AVH using MEG and coupled EEG/fMRI methods.

A potential limitation of this study relates to a failure to counter-balance button assignments across patients (all used the right button to trigger AVH onset and the left for AVH offset). It is possible that the laterality of some of our observations were influenced by this, although the laterality profiles reported herein are fully consistent with prior reports in the literature. The second weakness of the present analyses is that the majority of subjects in this study reported a history of hallucinations in multiple modalities, with some (*N* = 4) also reporting visual hallucinations during scanning. Analyses do not indicate that the presence of visual hallucinations modified network profiles with respect to AVH, but the study was likely underpowered to explore this. Future studies should provide a means for separate capture of visual hallucinations or auditory–visual combined hallucinations. Third, in considering the present results, it should be kept in mind that patients with schizophrenia and schizoaffective disorder were both included. On the one hand, analyses did not reveal any differences in activity profiles between the groups and there is not an *a priori* reason to anticipate differences, but the study was insufficiently powered to identify anything below a very large effect size.

Overall, the data support the initial hypothesis that coordinated activity in auditory and language cortical areas is a fundamental correlate of AVH. However, based on our own GLM data and that of others, it was anticipated that inferior frontal (rather than posterior temporal) language regions would be of greatest import. This was not borne out by the current study. Future work should, therefore, explore if there is a need for the identified ACPLN network to interact with other networks (e.g., inferior frontal or hippocampal networks) in the generation of AVH.

## Author Contributions

RT and JT are the principal investigators on the project and were responsible for all phases of the project, from conceptualization to publication. CC and CW performed the fMRI data analyses under the supervision of JT and VC. JL, JS, and VC worked on all phases of this project and were particularly involved in readying the manuscript for publication. JB oversaw clinical diagnostic aspects of the research and worked with RT on development of the clinical protocols. JH and RB helped to develop the neuroimaging data collection protocols and were responsible for management and organization of all data presented. JF was involved in project conceptualization, design, and editorial revision of the manuscript. All authors participated in development of the manuscript.

## Conflict of Interest Statement

The authors declare that the research was conducted in the absence of any commercial or financial relationships that could be construed as a potential conflict of interest.

## Footnotes

^1^www.neurobs.com

^2^www.fil.ion.ucl.ac.uk/spm/software/spm5

^3^http://www2.warwick.ac.uk/fac/sci/statistics/staff/academic-research/nichols/software/snpm

^4^http://mialab.mrn.org/software/gift/index.html

^5^Lewine JD, Thoma RJ, Canive JM, Davis JT. Neuromagnetic correlates of auditory hallucinations in schizophrenia: preliminary findings. *Neuropsychiatr Electrophysiol* (under review).
